# Consecutive reference intervals for biochemical indices related to serum lipid levels and renal function during normal pregnancy

**DOI:** 10.1186/s12884-022-04960-0

**Published:** 2022-08-15

**Authors:** Lina Wu, Qijun Wu, Qiang Li, Shuang Cao, Yue Zhang, Yong Liu, Xiaosong Qin

**Affiliations:** 1 Department of Laboratory Medicine, Shengjing Hospital of China Medical University, No. 36, San Hao Street, Shenyang, Liaoning 110004 People’s Republic of China; 2 Liaoning Clinical Research Center for Laboratory Medicine, Shenyang, China; 3 Department of Clinical Epidemiology, Shengjing Hospital of China Medical University, Shenyang, China

**Keywords:** Reference interval, Pregnancy, Serum lipid, Renal function, Biochemical indices

## Abstract

**Background:**

Physiological changes that occur during pregnancy can influence serum lipid levels and laboratory tests for renal function. Therefore, we established consecutive and reliable RIs for serum lipid and renal function indices for pregnant women in China throughout the entirety of pregnancy.

**Methods:**

We included 120 healthy pregnant women who underwent a naturally conceived and uncomplicated pregnancy and delivered a healthy singleton neonate. Serum samples were collected at ten time points (pre-pregnancy, gestational age ≤ 8 weeks (W), 8 W^+1^ to 12 W, 12 W^+1^ to 16 W, 16 W^+1^ to 20 W, 20 W^+1^ to 24 W, 24 W^+1^ to 28 W, 28 W^+1^ to 32 W, 32 W^+1^ to 36 W, and 36 W^+1^ to 40 W) and analyzed for ten common serum lipid and renal function analytes. RIs were calculated according to the International Federation of Clinical Chemistry and Laboratory Medicine recommendations and compared with the established RIs for healthy adult women.

**Results:**

During pregnancy, we observed significant increases in total cholesterol (TC), triglycerides (TG), high density lipoprotein cholesterol (HDL-C), low density lipoprotein cholesterol (LDL-C), apolipoprotein-A1 (Apo-A1), apolipoprotein-B (Apo-B), cystatin C (Cys-C), and estimated glomerular filtration rate (eGFR). We also observed clear reductions in urea, creatinine (Crea), and uric acid (UA). Compared with the previously established RIs, the most significant misclassifications were recorded for TG, Apo-A1, Crea, and eGFR.

**Conclusions:**

We successfully described key changes in serum lipid levels and renal function indices throughout pregnancy. It is important to establish RIs for blood indices in women undergoing normal pregnancies during different period of pregnancy to avoid the misdiagnosis of disease states.

**Supplementary Information:**

The online version contains supplementary material available at 10.1186/s12884-022-04960-0.

## Introduction

Physiological alterations in organ function during a normal pregnancy can exert considerable effects on the results of many biochemical laboratory tests, thus leading to deviations from the established reference intervals (RIs) for healthy non-pregnant women. For example, the levels of total cholesterol (TC), triglyceride (TG), and low-density lipoprotein cholesterol (LDL-C) are known to increase during pregnancy as a result of increased hepatic synthesis and the reduced activity of lipoprotein lipase [1, 2]. Pregnancy-induced physiological changes in the kidney mainly include increased renal plasma flow and glomerular filtration rate (GFR) [1, 2]. Measures of renal function, including serum creatinine (Crea), urea, and uric acid (UA), are known to fall during the first trimester due to the elevation of GFR, but then remain steady in the second trimester, and increase towards pre-pregnancy levels during the third trimester [1, 3, 4]. Therefore, it is vital that we establish gestational age-special RIs for women with normal pregnancies to distinguish between normal physiological changes and pathological conditions.

Previous studies have reported some region-specific RIs for clinical chemistry tests for healthy pregnant women during different stages of pregnancy [5–8]. For example, a cross-sectional study, carried out in Zhengzhou city, China, divided 13,656 healthy pregnant women into five gestational age groups, analyzed the results of blood biochemistry tests in different groups, and reported gestational age-specific RIs for 15 biochemical substances related to hepatic and renal function [7]. However, most of these studies did not recruit the same group of women to perform consecutive laboratory tests throughout the entire period of pregnancy. Very few studies have collected blood samples from a consecutive study population; moreover, these studies only involved small sample sizes [6, 9].

Given this lack of consecutive analysis, and the clear heterogeneity evident in previous study populations, we aimed to establish consecutive and reliable RIs for pregnant women in China by analyzing the results of biochemical indicators related to lipid metabolism and renal function within the same group of healthy pregnant women from pre-pregnancy and throughout the entire duration of pregnancy.

## Materials and methods

### Study population

**Fig. 1:**
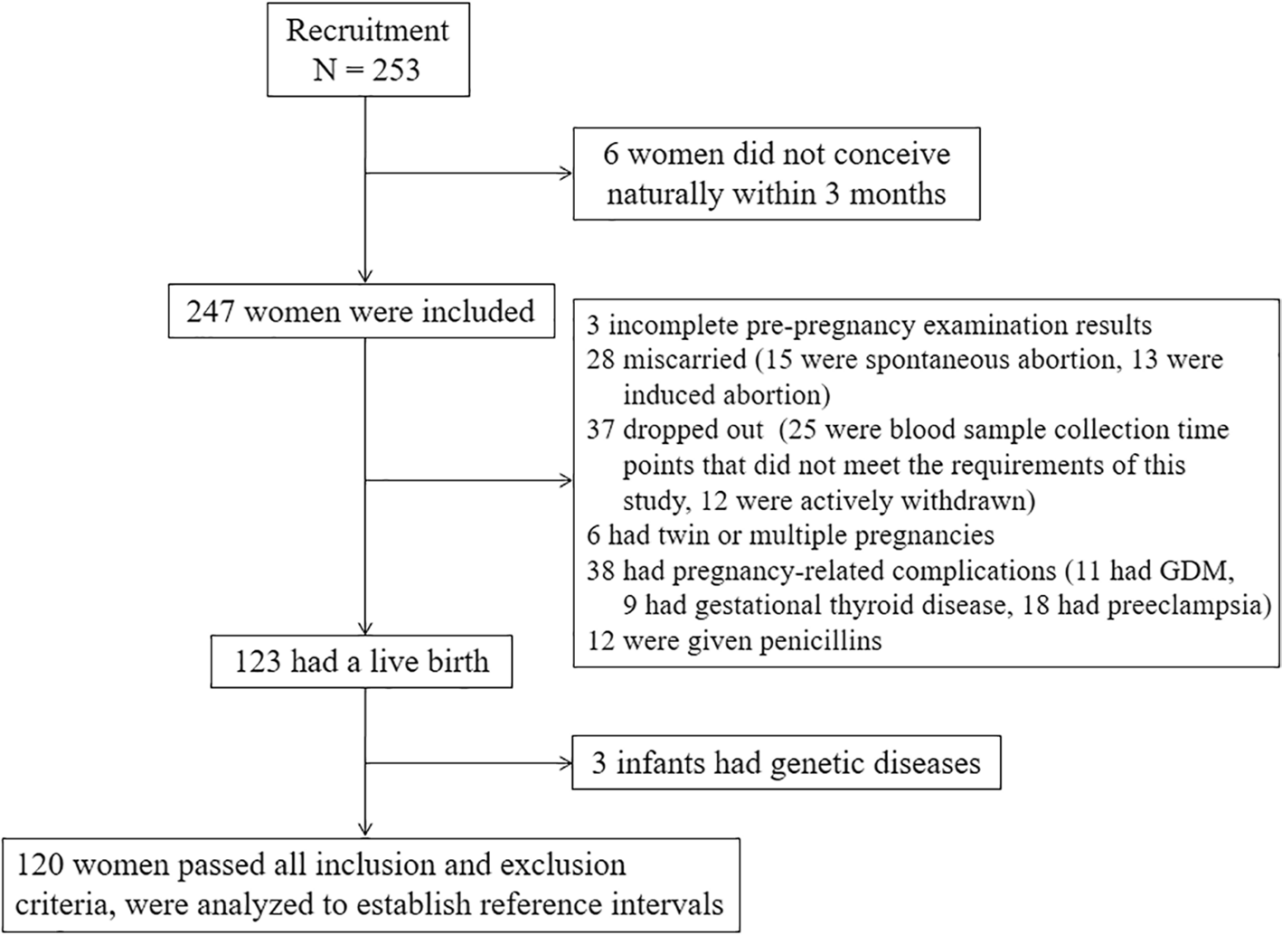
Flow chart of the selection of participants

This was a prospective cohort study, carried out between October 2016 and April 2018. We recruited all women of childbearing age who completed pre-pregnancy examinations, had recent pregnancy intentions, and volunteered to participate in this study at the Obstetrics Clinic at Shengjing Hospital of China Medical University. As seen in Fig. 1, 253 women of child bearing age expressed an interest in participating in this study; of these, 247 were included in the final analysis (six women were excluded because they did not conceive naturally within three months or lost interest in the study). Based on this cohort, we carried out a longitudinal study to investigate the changes in blood indicators from pre-pregnancy to delivery. All participants received oral and written information relating to the study prior to participation and signed an informed consent form according to the standards of the Helsinki declaration. The study was approved by the Ethics Committee of Shengjing Hospital of China Medical University (Ethical reference number: 2017PS264K).

#### Inclusion criteria

Subjects were included if they met the following criteria: (1) aged at least 18 years; (2) received normal laboratory reports prior to pregnancy (liver, kidney, and thyroid function tests; serum lipids; serum glucose; routine urine tests; human immunodeficiency virus antibody; syphilis antibody; hepatitis A antibody; hepatitis B serological markers; and hepatitis C antibodies); (3) conceived naturally within three months after pre-pregnancy examinations, devoid of pregnancy-related diseases, and accepted venous blood collection in strict accordance with birth cohort standards; and (4) fetal ultrasound examination was normal, and the newborn had no congenital diseases during one year of follow-up after birth.

#### Exclusion criteria

Exclusion criteria were determined in accordance with ‘Defining, Establishing, and Verifying Reference Intervals’ in the Clinical Laboratory; Approved Guidelines (Third edition) [10]. Subjects were excluded for the following reasons: (1) excessive drinking (average alcohol consumption > 30 g/day) or smoking (> 20 cigarettes/day), or the intake of drugs before and/or during pregnancy; (2) hypertension (systolic blood pressure > 140 mmHg and diastolic blood pressure > 90 mmHg) before and/or during pregnancy; (3) body mass index (BMI) ≥ 28 kg/m^2^ or ≤ 8.5 kg/m^2^ before pregnancy; (4) blood transfusion or blood donation within 6 months of pregnancy, or surgery within 4 months before pregnancy; (5) a history of recurrent pregnancy loss (≥ 3 consecutive losses), all types of fertility treatments, known or initially diagnosed abnormalities of the uterus or tubes, or ongoing drug abuse; (6) the use of medication within 2 weeks of blood collection; (7) diagnosed with cancer, impaired kidney function, diseases of the hepatic, endocrine, or cardiovascular systems, or other chronic diseases before or during pregnancy; or diagnosed acute illnesses during pregnancy; (8) a history of genetic disease; (9) complex pregnancies, such as twin or multiple pregnancies; or (10) low birth weight or preterm infants and 5 min Apgar scores < 7.

As shown in Fig. 1, at the end of follow-up, after excluding 127 participants, we included 120 women for the establishment of RIs.

### Estimation of gestational age

All participants received transvaginal ultrasonography (GE Voluson E10, GE Healthcare, Solingen, Germany) at 7–12 weeks of pregnancy to accurately calculate the gestational age. The calculation of gestational age was based on the following formula: gestational age = crown-rump-length (cm) + 6.5 [11].

### Blood sample collection

For each of the enrolled pregnant women, we collected 3 mL of fasting venous blood at ten time points (pre-pregnancy, gestational age ≤ 8 W, 8 W^+1^ to 12 W, 12 W^+1^ to 16 W, 16 W^+1^ to 20 W, 20 W^+1^ to 24 W, 24 W^+1^ to 28 W, 28 W^+1^ to 32 W, 32 W^+1^ to 36 W, and 36 W^+1^ to 40 W). During pregnancy, the time interval between two adjacent time points for blood collection was 4 weeks ± 2 days. Immediately after collection, blood samples were placed into serum separation tubes and centrifuged at 3500 g for 10 min. The samples were used in laboratory tests immediately thereafter. All testing indicators were determined within the stable period of preservation. The remaining serum was stored at − 70 °C to await further analysis.

### Laboratory analysis

Levels of TC, TG, high density lipoprotein cholesterol(HDL-C), LDL-C, apolipoprotein-A1 (Apo-A1), Apo-B, urea, Crea, Cys-C, and UA were determined in the serum samples using an Architect C16000 automatic biochemistry analyzer (Name of Manufacturer: Abbott Laboratories; Production Address of Manufacturer: 1385, Shimoishigami, Otawara-shi, Tochigi 324–8550, Japan; Registered Address of manufacturer: 100/200 Abbott Park Road Abbott Park, Illinois, 60,064 USA). The estimated GFR (eGFR) was calculated using the equations from the Chronic Kidney Disease Epidemiology Collaboration for Asians [12]. The analyzed parameters, with abbreviations, method characteristics, assay coefficients of variation, and traceability data, are listed in Table 1.

**Table 1:** Performed tests, methods and analytical details

Analyte (abbreviation)	Method	CV%	Traceability
Total cholesterol (TC)	Cholesterol oxidase	0.91	NIST 911
Triglycerides (TG)	Dissociated glycerine	1.01	NIST Glyceryl trioleate
Low density lipoprotein cholesterol (LDL-C)	Selective solubility	3.72	NIST 911
High density lipoprotein cholesterol (HDL-C)	PEG modified enzyme	1.34	NIST 911
Apolipoprotein A1 (Apo-A1)	Immune transmission turbidimetry	1.11	WHO SP1-01
Apolipoprotein B (Apo-B)	Immune transmission turbidimetry	1.44	WHO SP3-07
Urea	Urease colorimetry	1.65	NIST SRM 912
Creatinine (Crea)	Enzymatic method	0.89	NIST SRM 914a
Cystatin C (Cys-C)	Latex immunoturbidimetry	2.99	GBW(E) 090,437
Uric acid (UA)	Uricase colorimetry	0.77	NIST SRM 913

The total analytical imprecision for the experimental method used to calculate the reference intervals is given for each assay as an averagevariation coefficient (CV%) of two concentrations of internal controls through one year. Samples with preanalytic handling time longer than the stabilityin the blood (in days) at room temperature were removed from the calculations

### Statistical analysis

Statistical analyses were performed using SPSS version 26.0 (SPSS Inc., Chicago, IL USA). The Kolmogorov–Smirnov test was used to analyze the normality of the quantitative data. Quantitative data that followed a normal distribution were expressed as mean ± standard deviation, and that did not follow a normal distribution were expressed as median (with inter-quartile range). According to Clinical Laboratory Standards Institute document C28-A3, differences in serum lipid levels and renal function indices between groups were analyzed using the Kruskal–Wallis test with Dunn post hoc tests (for continuous variables, R package FSA) [10]. RIs (2.5^th^ and 97.5^th^ percentiles for two-sided tests, 95^th^ percentiles for one-sided tests) with 90% confidence intervals (CIs) for different gestational periods were calculated. The non-parametric bootstrap method with 500 iterations was used in accordance with the International Federation of Clinical Chemistry and Laboratory Medicine (IFCC) recommendations [13]. The one-way ANOVA followed by Bonferroni adjustment was used to compare difference of BMI in each stages, and Spearman’s rank correlation test was used to assess the relationship between BMI and weight gain in pregnancy. *P* < 0.05 was considered statistically significant.

## Results

The mean age of participants was 30 ± 3 years, and the mean BMI was 21.14 ± 2.63 kg/m^2^ pre-pregnancy. The BMI during pregnancy was significantly larger than that before pregnancy (all *P* < 0.05). The distributions and comparative analysis of BMI, serum lipid levels, and renal function indices, between pre-pregnancy and different gestational periods are presented in Table 2. Compared with the pre-pregnancy values, most of the parameters for each gestational age were significantly different. Only a few test results did not change significantly when compared between pre-pregnancy and different gestational ages. The correlation relationships between BMI, weight gain in pregnancy and serum lipid level are listed in supplemental Table 1. Level of serum TG was positively correlated with BMI and weight gain during pregnancy. Level of serum HDL-C was inversely correlated with BMI at ≤ 24 weeks of gestation. The RIs for each gestational period are shown in Table 3 alongside the general established intervals. The laboratory used reference values for healthy females that were derived from WS/T 410–2013, WS/T 404.5–2015, WS/T 463–2015 (Ministry of Health PRC), and local recommendations [14–16].

**Table 2:** Results of serum lipid level and renal function indexes in normal pregnant women during different stages of pregnancies

Index	pre-pregnancy	Gestational age								
		** ≤ 8 W**	**8 W**^**+1**^**-12 W**	**12 W**^**+1**^**-16 W**	**16 W**^**+1**^**-20 W**	**20 W**^**+1**^**-24 W**	**24 W**^**+1**^**-28 W**	**28 W**^**+1**^**-32 W**	**32 W**^**+1**^**-36 W**	**36 W**^**+1**^**-40 W**
**BMI** (kg/m^2^)^a^	21.14 ± 2.63	21.92 ± 2.99	21.71 ± 4.20	21.95 ± 4.30	22.10 ± 5.14*	23.34 ± 4.37*	24.27 ± 4.46*	25.16 ± 4.57*	26.03 ± 4.65*	26.70 ± 4.78*
**TC** (mmol/L)^b^	4.42 (4.17, 4.74)	3.92 (3.63, 4.35)*	4.23 (3.79, 4.62)	4.69 (4.24, 5.10)	5.06 (4.54, 5.68)*	5.62 (5.06, 6.25)*	6.04 (5.32, 6.60)*	6.39 (5.61, 7.06)*	6.45 (5.55, 7.00)*	6.50 (5.73, 7.44)*
**TG** (mmol/L)^b^	0.86 (0.65, 1.22)	0.72 (0.56, 0.87)	1.06 (0.82, 1.35)	1.43 (1.11, 1.77)*	1.73 (1.32, 2.11)*	2.02 (1.56, 2.41)*	2.28 (1.85, 2.75)*	2.63 (2.04, 3.19)*	2.98 (2.54, 3.54)*	3.23 (2.70, 4.02)*
**HDL-C**(mmol/L)^b^	1.43 (1.24, 1.61)	1.42 (1.23, 1.66)	1.60 (1.44, 1.84)*	1.79 (1.57, 2.04)*	1.92 (1.71, 2.17)*	2.06 (1.75, 2.32)*	2.16 (1.90, 2.37)*	2.05 (1.79, 2.32)*	1.96 (1.73, 2.29)*	2.02 (1.74, 2.32)*
**LDL-C**(mmol/L)^b^	2.55 (2.33, 2.82)	2.17 (1.83, 2.58)*	2.15 (1.86, 2.49)*	2.41 (2.06, 2.76)	2.62 (2.24, 3.06)	3.00 (2.59, 3.60)*	3.31 (2.82, 3.78)*	3.63 (3.01, 4.18)*	3.59 (3.03, 4.26)*	3.59 (2.95, 4.31)*
**Apo-A1** (g/L)^b^	1.48 (1.36, 1.60)	1.52 (1.37, 1.67)	1.78 (1.58, 1.97)*	2.03 (1.84, 2.15)*	2.14 (1.97, 2.26)*	2.23 (2.03, 2.34)*	2.24 (2.13, 2.36)*	2.25 (2.13, 2.38)*	2.22 (2.09,2.34)*	2.28 (2.12, 2.40)*
**Apo-B** (g/L)^b^	0.86 (0.79, 0.93)	0.66 (0.57, 0.78)*	0.68 (0.57, 0.79)*	0.78 (0.70, 0.92)	0.88 (0.74, 1.03)	0.99 (0.86, 1.17)*	1.08 (0.96, 1.27)*	1.19 (1.01, 1.41)*	1.20 (1.04, 1.42)*	1.22 (1.05, 1.48)*
**Urea** (mmol/L)^b^	4.19 (3.64, 5.06)	2.99 (2.58, 3.51)*	2.53 (2.27, 2.92)*	2.50 (2.13, 2.92)*	2.51 (2.14, 2.90)*	2.60 (2.23, 3.00)*	2.67 (2.36, 3.04)*	2.66 (2.30, 3.14)*	2.68 (2.28, 3.06)*	2.94 (2.47, 3.46)*
**Crea** (μmol/L)^b^	57.4 (53.7, 61.1)	46.4 (43.0, 51.9)*	41.9 (39.5, 46.1)*	40.4 (38.1, 44.5)*	40.2 (37.3, 44.1)*	39.9 (37.2, 43.5)*	40.5 (37.7, 43.7)*	41.4 (38.3, 45.7)*	42.4 (38.4, 46.1)*	45.2 (41.7, 50.1)*
**Cys-C** (mg/L)^b^	0.73 (0.68, 0.79)	0.75 (0.71, 0.81)	0.70 (0.64, 0.77)*	0.71 (0.66, 0.78)	0.76 (0.69, 0.81)	0.79 (0.73,0.88)*	0.86 (0.76, 0.93)*	0.94 (0.86, 1.04)*	1.08 (0.97, 1.23)*	1.25 (1.14, 1.39)*
**UA** (μmol/L)^b^	278 (242, 313)	225 (193, 259)*	201 (174, 231)*	207 (193, 235)*	215 (187, 237)*	219 (193, 255)*	227 (201, 252)*	235 (208, 270)*	256 (223, 288)*	281 (245. 321)
**eGFR** [mL/(min·1.73m^2^)]^b^	119 (116, 122)	127 (123, 131)*	132 (128, 136)*	134 (129, 137)*	134 (130, 137)*	135 (131, 138)*	134 (129, 138)*	133 (129, 137)*	132 (127, 137)*	129 (124, 133)*

*Apo-A1* Apolipoprotein-A1, *Apo-B* Apolipoprotein-B, *BMI* Body mass index, *Crea* Creatinine, *Cys-C* Cystatin C, *HDL-C* High density lipoprotein cholesterol, *LDL-C* Low density lipoprotein cholesterol, *TC* Total cholesterol, *TG* Triglycerides, *UA* Uric acid, *eGFR* Estimated glomerular filtration rate, *W* Week

Before and during pregnancy, the datas of BMI, TC, HDL-C followed a normal distribution and were expressed as mean ± standard deviation; TG, LDL-C, Apo-A1, Apo-B, Urea, Crea, Cys-C, UA did not follow a normal distribution and were expressed as median (interquartile range)

^a^Comparisons using the one-way ANOVA followed by Bonferroni adjustment

^b^Comparisons using the Kruskal–Wallis test followed by the Dunn post-test

^*^P < 0.05 was considered statistically significant, *p*-Value that indicates statistical comparisons for this study between the results for pre-pregnancy and each different stages of pregnancies

**Table 3:** Reference intervals for normal pregnant women in different stages of pregnancies

Analyte	Established interval	Gestational age									
		** ≤ 8 W**	**8 W**^**+1**^**-12 W**		**12 W**^**+1**^**-16 W**	**16 W**^**+1**^**-20 W**	**20 W**^**+1**^**-24 W**	**24 W**^**+1**^**-28 W**	**28 W**^**+1**^**-32 W**	**32 W**^**+1**^**-36 W**	**36 W**^**+1**^**-40 W**
**TC** (mmol/L)											
Mean	3.36–5.69	4.02	4.19		4.67	5.12	5.66	6.03	6.33	6.38	6.60
Lower limit		2.82 (2.68–3.01)	3.10 (2.88–3.25)		3.41 (3.28–3.62)	3.82 (3.46–4.05)	3.97 (3.15–4.25)	4.28 (3.78–4.56)	4.55 (4.14–4.77)	4.57 (4.46–4.66)	4.58 (4.28–4.98)
Upper limit		5.25 (5.02–6.62)	5.30 (5.15–5.77)		6.08 (5.71–6.32)	6.86 (6.49–7.02)	7.40 (7.11–7.61)	8.15 (7.62–8.43)	8.10 (7.91–8.48)	8.42 (8.00–8.90)	9.23 (8.25–10.97)
n%		14.2	5.8		4.2	20.0	45.0	60.0	70.8	69.2	75.8
**TG** (mmol/L)											
Median	0.40–1.69	0.72	1.06		1.43	1.73	2.02	2.28	2.63	2.98	3.23
Lower limit		0.34 (0.33–0.40)	0.57 (0.44–0.64)		0.81 (0.75–0.88)	0.82 (0.68–1.03)	1.00 (0.94–1.10)	1.23 (1.15–1.32)	1.35 (1.29–1.58)	1.67 (1.59–1.90)	2.02 (1.56–2.14)
Upper limit		2.16 (1.62–2.33)	2.30 (1.96–3.37)		3.08 (2.41–3.31)	3.99 (3.01–5.16)	4.28 (3.13–5.85)	4.86 (4.13–5.27)	5.76 (4.90–8.03)	6.46 (5.73–6.97)	6.27 (6.01–7.69)
n%		6.7	5.0		30.0	50.8	63.3	79.2	88.3	95.0	97.5
**HDL-C** (mmol/L)											
Mean	1.00–2.10	1.45	1.63		1.81	1.93	2.04	2.10	2.04	1.98	2.03
Lower limit		0.91 (0.73–0.98)	1.04 (0.86–1.10)		1.15 (0.94–1.26)	1.32 (1.14–1.35)	1.26 (1.19–1.35)	1.37 (1.06–1.49)	1.29 (1.08–1.51)	1.23 (0.85–1.36)	1.19 (0.94–1.37)
Upper limit		2.28 (1.97–2.44)	2.38 (2.19–2.44)		2.56 (2.48–2.63)	2.62 (2.49–2.78)	2.64 (2.59–2.83)	2.68 (2.56–3.32)	2.69 (2.59–2.95)	2.71 (2.56–3.45)	2.95 (2.55–3.98)
n%		5.8	6.7		15.0	39.0	41.7	52.5	43.3	35.0	37.5
**LDL-C** (mmol/L)											
Median	< 3.37	2.17		2.15	2.41	2.62	3.00	3.31	3.63	3.59	3.59
Upper limit		3.17 (3.03–4.41)		3.03 (2.74–3.12)	3.19 (3.13–3.63)	3.89 (3.61–4.23)	4.40 (4.10–4.49)	4.94 (4.42–5.40)	5.00 (4.87–5.18)	5.16 (4.85–5.56)	5.56 (5.08–6.05)
n%		0.0		0.0	0.0	9.2	28.3	40.8	59.2	54.2	55.8
**Apo-A1** ( g/L)											
Median	1.20–1.90	1.52	1.78		2.03	2.14	2.23	2.24	2.25	2.22	2.28
Lower limit		1.00 (0.93–1.15)	1.28 (1.08–1.37)		1.38 (1.24–1.55)	1.60 (1.32–1.68)	1.61 (1.40–1.75)	1.73 (1.39–1.80)	1.68 (1.53–1.74)	1.63 (1.28–1.83)	1.82 (1.36–1.85)
Upper limit		2.11 (1.87–2.24)	2.30 (2.12–2.33)		2.42 (2.37–2.49)	2.43 (2.39–2.62)	2.58 (2.55–2.84)	2.87 (2.71–2.99)	2.77 (2.61–3.50)	2.91 (2.69–3.10)	2.85 (2.64–3.00)
n%		3.3	31.7		65.0	80.8	87.5	90.0	90.0	90.0	91.7
**Apo-B** ( g/L)											
Median	0.75–1.50	0.66		0.68	0.78	0.88	0.99	1.08	1.19	1.20	1.22
Lower limit		0.40 (0.39–0.46)		0.44 (0.32–0.46)	0.48 (0.40–0.54)	0.53 (0.43–0.58)	0.61 (0.45–0.69)	0.64 (0.56–0.73)	0.74 (0.69–0.79)	0.78 (0.69–0.83)	0.78 (0.71–0.83)
Upper limit		1.16 (0.99–1.41)		1.00 (0.92–1.22)	1.12 (1.08–1.32)	1.37 (1.29–1.42)	1.51 (1.39–1.61)	1.58 (1.52–1.81)	1.66 (1.64–1.75)	1.71 (1.67–1.98)	1.94 (1.63–2.22)
n%		67.5		63.3	37.5	25.0	9.2	10.0	13.3	14.2	20.0
**Urea** (mmol/L)											
Median	2.60–7.50	2.99	2.53		2.50	2.51	2.60	2.67	2.66	2.68	2.94
Lower limit		1.97 (1.79–2.08)	1.75 (0.60–1.81)		1.57 (1.31–1.75)	1.51 (1.18–1.63)	1.60 (1.34–1.89)	1.71 (1.51–1.97)	1.68 (1.59–1.79)	1.50 (1.35–1.77)	1.91 (1.89–2.04)
Upper limit		4.73 (4.49–6.61)	3.83 (3.54–5.05)		4.11 (3.67–4.56)	4.04 (3.37–4.85)	3.87 (3.65–4.20)	4.55 (3.82–4.72)	3.98 (3.82–4.07)	4.29 (3.88–5.04)	4.77 (4.05–5.34)
n%		25.0	52.5		58.3	56.7	48.3	39.2	43.3	43.3	29.2
**Crea** (μmol/L)											
Median	41.0–73.0	46.4	41.9		40.4	40.2	39.9	40.5	41.4	42.4	45.2
Lower limit		37.1 (30.8–38.1)	33.2 (31.8–34.9)		32.1 (28.4–33.0)	30.0 (28.3–31.8)	28.3 (26.4–32.6)	30.3 (26.9–32.1)	28.1 (27.3–32.1)	29.3 (27.4–33.6)	34.4 (33.4–35.3)
Upper limit		62.4 (58.8–64.0)	54.9 (52.8–57.0)		52.7 (50.4–56.0)	51.9 (50.2–54.8)	52.5 (49.2–64.9)	55.8 (52.7–63.2)	55.4 (52.5–61.7)	55.6 (52.7–63.5)	59.8 (57.4–63.7)
n%		10.8	40.8		52.5	56.7	59.2	51.7	46.7	40.8	20.8
**Cys-C** ( mg/L)											
Median	0.59–1.03	0.75	0.70		0.71	0.76	0.79	0.86	0.94	1.08	1.25
Lower limit		0.63 (0.57–0.64)	0.55 (049–0.57)		0.57 (0.51–0.61)	0.59 (0.56–0.62)	0.62 (0.58–0.64)	0.64 (0.60–0.67)	0.73 (0.69–0.75)	0.82 (0.77–0.85)	0.96 (0.88–0.97)
Upper limit		0.95 (0.91–0.97)	0.86 (0.83–0.96)		0.88 (0.85–1.05)	0.95 (0.92–0.99)	1.03 (0.98–1.10)	1.07 (1.05–1.14)	1.33 (1.23–1.41)	1.68 (1.51–1.76)	1.72 (1.68–1.96)
n%		0.0	5.8		0.8	0.0	0.0	5.0	23.3	60.0	85.8
**UA** (μmol/L)											
Median	150–350	225	201		207	215	219	227	235	256	281
Lower limit		145 (98–157)	133 (105–143)		141 (111–150)	141 (115–154)	150 (98–162)	142 (106–162)	145 (108–165)	161 (146–175)	167 (128–188)
Upper limit		326 (320–343)	299 (277–315)		308 (273–327)	325 (306–375)	326 (299–338)	318 (309–408)	331 (321–363)	355 (343–412)	464 (406–525)
n%		2.5	5.8		3.3	2.5	0.0	1.7	3.3	0.8	9.2
**eGFR** [mL/(min·1.73m^2^)]											
Median	80–120	127	132		134	134	135	134	133	132	129
Lower limit		114 (112–116)	120 (119–122)		122 (117–124)	121 (119–123)	119 (112–124)	117 (115–121)	118 (117–121)	117 (115–120)	117 (109–118)
Upper limit		142 (139–148)	144 (142–148)		147 (143–152)	148 (145–151)	153 (147–156)	152 (143–155)	154 (146–157)	149 (142–154)	144 (141–146)
n%		83.3	95.8		95.0	95.8	95.0	94.2	96.2	93.3	90.0

*Apo-A1* Apolipoprotein-A1, *Apo-B* Apolipoprotein-B, *Crea* Creatinine, *Cys-C* Cystatin C, *HDL-C* High density lipoprotein cholesterol, *LDL-C* Low density lipoprotein cholesterol, *TC* Total cholesterol, *TG* Triglycerides, *UA* Uric acid, *eGFR* Estimated glomerular filtration rate, *W* Week

The 90% confidence intervals for the lower and upper limits are given in parentheses;

n%: proportions of pregnant women whose parameters values were outside the range of established reference intervals for healthy adult females

### Changes and RIs for serum lipid level

**Fig. 2:**
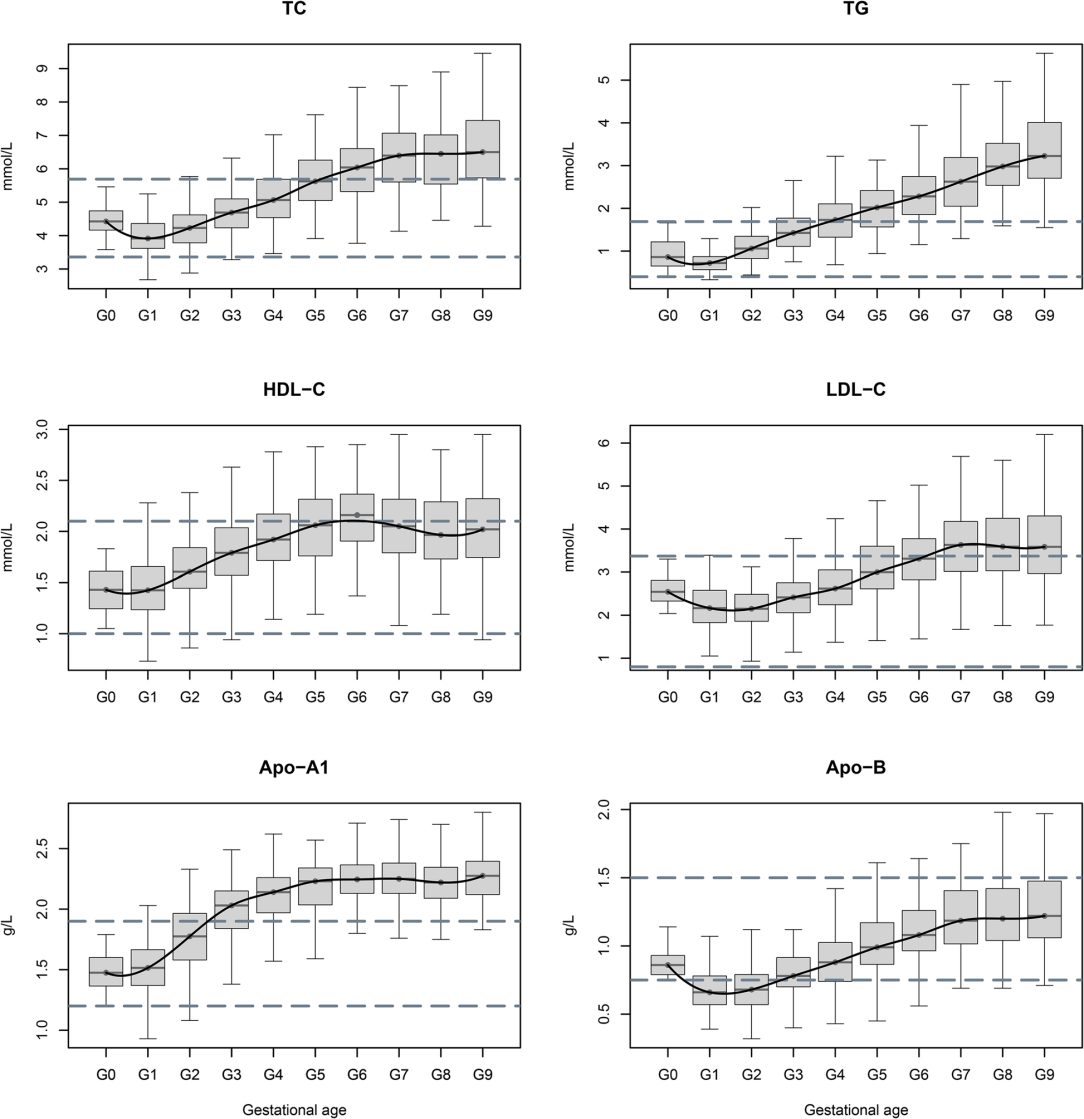
Distributions and changes of serum lipid levels. The ranges between the two dotted lines represent non-pregnant normal reference interval. Apo-A1, apolipoprotein-A1, Apo-B, apolipoprotein-B, G0: pre-pregnancy, G1: ≤ 8 W, G2: 8 W^+1^-12 W, G3: 12 W^+1^-16 W, G4: 16 W^+1^-20 W, G5: 20 W^+1^-24 W, G6: 24 W^+1^-28 W, G7: 28 W^+1^-32 W, G8: 32 W^+1^-36 W, G9: 36 W^+1^-40 W, HDL-C: high density lipoprotein cholesterol, LDL-C: low density lipoprotein cholesterol, TC: total cholesterol, TG: triglycerides

The trend for changes in serum lipid level are shown in Fig. 2. The concentrations of TC decreased during the first trimester but then increased until delivery. Median TC values were significantly lower during pre-pregnancy when compared to the second and third trimesters. TC levels reached peak values at 36^+1^ to 40 weeks of gestation (RI: 4.58–9.23 mmol/L); the mean TC level at 36^+1^ to 40 weeks of gestation was more than 1.5-fold higher than that during pre-pregnancy.

TG showed a slight reduction at ≤ 8 weeks of gestation; the median value was lower that during pre-pregnancy. Levels of TG then increased rapidly until delivery; TG was significantly higher after only 12 weeks of gestation. The most significant misclassification occurred during the third trimester, as the upper reference limit for our results was threefold higher than the upper reference limit for healthy adult women; this could cause more than 90% of pregnant women to be excluded.

HDL-C and Apo-A1 showed a similar trend, with concentrations beginning to become apparent at 8 weeks of gestation and continuing to rise until delivery. The peak concentrations of HDL-C and Apo-A1 were approximately 1.5-fold higher than the pre-pregnancy level. The misclassification rate for Apo-A1 was approximately 90% in the second and third trimesters.

The concentration of LDL-C remained within conventional limits at ≤ 16 weeks, but then increased until delivery. LDL-C levels peaked at 36^+1^ to 40 weeks of gestation; the median value was approximately 1.5-fold higher than the pre-pregnancy level.

Apo-B levels were low prior to pregnancy but then increased slowly during pregnancy. Levels of Apo-B in the first trimester were significantly lower than those during pre-pregnancy, resulting in a misclassification rate of approximately 60%. Apo-B levels in the second trimester were similar to those prior to pregnancy and were slightly higher in the third trimester than prior to pregnancy.

### Changes and RIs for indicators of renal function

**Fig. 3:**
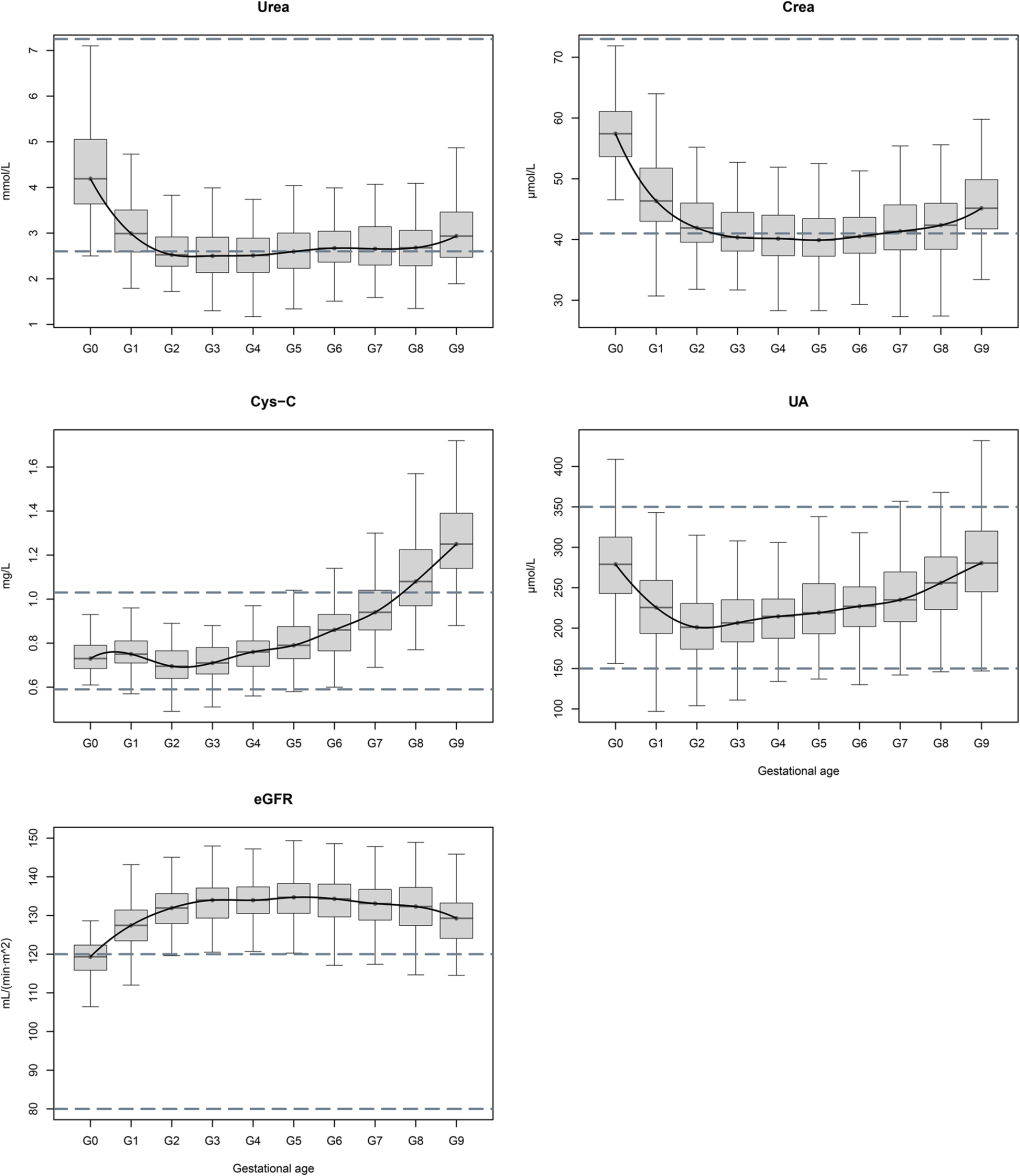
Distributions and changes of renal function indexes. The ranges between the two dotted lines represent non-pregnant normal reference interval. Crea: creatinine, Cys-C: cystatin C, eGFR: estimated glomerular filtration rate, G0: pre-pregnancy, G1: ≤ 8 W, G2: 8 W^+1^-12 W, G3: 12 W^+1^-16 W, G4: 16 W^+1^-20 W, G5: 20 W^+1^-24 W, G6: 24 W^+1^-28 W, G7: 28 W^+1^-32 W, G8: 32 W^+1^-36 W, G9: 36 W^+1^-40 W, UA: uric acid

The trends for change in renal function indicators are shown in Fig. 3. Urea and Crea showed a similar trend for change; values throughout pregnancy were significantly lower than those in pre-pregnancy. Both the levels of Urea and Crea fell significantly during the first trimester, and then remained relatively stable during the second trimester. Urea fell to the lowest level at 12^+1^ to 16 weeks; this was approximately half that of the pre-pregnancy level. Crea fell to the lowest level at 20^+1^ to 24 weeks; this was approximately two-thirds of the pre-pregnancy level. Both indices subsequently increased slightly, although the indices were still lower than the conventional intervals in the third trimester.

Cys-C decreased gradually during the first trimester and reached the lowest level at 8^+1^ to 12 weeks. After that, the level gradually increased and peaked at 36^+1^ to 40 weeks; this could have led to a misclassification rate of 85.8%.

UA fell significantly during the first trimester, and dropped to its lowest value at 8^+1^ to 12 weeks; this was approximately three quarters of the pre-pregnancy level. UA then increased slightly during the second and third trimesters. Compared with conventional limits, the misclassification rates were less than 10% during the entire pregnancy.

The level of eGFR showed a slight increase at ≤ 8 weeks of gestation, and then rose to a median level of about 130 mL/(min·1.73m^2^) and remain stable. The level of eGFR decreased slightly at 32^+1^ to 40 weeks, but not significantly. This could cause more than 90% of pregnant women to be excluded during 8^+1^ to 40 weeks.

## Discussion

Blood tests are often required throughout pregnancy to identify pregnancy-associated complications that may be harmful to pregnant women or fetuses. Correct and appropriate RIs for pregnancy are vital for supporting clinical decisions. This study successfully established detailed RIs for ten laboratory blood tests across different gestational ages during the entire pregnancies of women who conceived naturally and experienced an uncomplicated pregnancy with the delivery of a healthy singleton neonate. This made it possible for us to assess how these analytes deviated from the established RIs of non-pregnant women.

Under the influence of altered endogenous synthesis, transport mechanisms, and sex hormone concentrations, serum lipid levels usually undergo a series of physiological changes during pregnancy [17–19]. The changes in lipid metabolism during pregnancy are characterized by fat accumulation and increased tissue lipolysis, which are physiologically necessary during pregnancy [20]. However, maternal lipid metabolism disorders have been shown to be associated with an increased risk of multiple adverse pregnancy outcomes, including gestational diabetes mellitus, gestational hypertension, preeclampsia, and preterm delivery [21, 22]. Our present results showed that serum lipid levels increased to varying degrees during pregnancy, with TG levels showing the most significant changes. These results were consistent with the trend for serum lipid concentration changes reported in most previous studies [5, 8, 23].

The RIs for TC and TG increased significantly from the second trimester to delivery, especially for TG; findings which were consistent with those reported by previous studies that were conducted in Denmark and China [8, 23].Using the established reference intervals for healthy adult women, the overall percentage of out-of-range TG values was approximately 93.6% in the third trimester.TC is used for fetal cell membrane construction and acts as a precursor for bile acids and steroid hormones. TG is the energy depot for maternal dietary fatty acids [24]. Both TC and TG play an important role in the growth and development of the fetus. However, our RIs for TG were significantly higher in the third trimester than the RIs in a previous study of Caucasian women that was carried out in Denmark [8] and may be due to differences in diet and environmental factors. To meet the increased demand for sex steroids, the levels of TC, HDL-C, and LDL-C all increase during pregnancy; this is because the levels of precursor substrates also increase during pregnancy [2]. In the study, the concentrations of HDL-C and LDL-C did not increase significantly during the first trimester but began to increase slowly thereafter to reach a peak before delivery; the levels at delivery were 1.5-fold higher than those prior to pregnancy. HDL-C and LDL-C also increased slightly during different periods of pregnancy, changes that were also reported by Friis et al. and Ying et al. [5, 23].Apo-A1 is necessary for normal HDL-C biosynthesis, while Apo-B is the main protein component of LDL-C and very low-density lipoprotein (VLDL) [25, 26]. Our data showed that the trends for Apo-A1 and Apo-B were similar to those of HDL-C and LDL-C. Furthermore, Larsson et al. indicated that the parallel increase in both Apo-A1 and Apo-B also reduced impact on the Apo-B/A1 ratio [6]. The 2019 ESC/EAS Guidelines refined and emphasized the importance of lipid modification to reduce cardiovascular risk, with particular attention to BMI, TG, LDL-C, and Apo-B control [27]. Although most of these indicators increased significantly during pregnancy, the ESC/EAS Guidelines still insist that no lipid-lowering drugs should be administered during pregnancy. Therefore, pregnant women should pay attention to controlling the growth of index levels to reduce the risk of cardiovascular diseases.

During normal pregnancy, the renal function of pregnant women also undergoes significant physiological changes. Due to the effect of progesterone, the renal calyceal and ureters of pregnant women undergo dilation and consequential changes in metabolism. Previous studies indicated that pregnancy is associated with changes in renal structure, an increase in blood volume, and the release of specific hormones, all of which could lead to changes in the GFR and the dilution of urea, Crea, Cys-C, and UA in the serum [28]. The results of this study suggest that the eGFR was at a high level throughout pregnancy. Urea, Crea, and UA are the most sensitive and appropriate indicators with which to measure renal function during pregnancy, and are all related to the number of gestational weeks [29]. By monitoring indicators throughout the entirety of pregnancy, we found that the levels of Urea and Crea decreased rapidly in the first trimester, but increased slowly from 36^+1^ to 40 weeks; these findings were similar to those reported by Dai et al. and Larsson et al. [6, 7]. This may be related to the reduction of renal plasma flow during the third trimester of pregnancy when compared with pre-pregnancy levels [1]. In addition, the levels of Cys-C decreased slightly during the first trimester, but then increased, particularly during the third trimester. This trend was consistent with a previous study (7) although the RIs for our data were higher [7]. The levels of UA significantly decreased during the first trimester, but then increased progressively with gestational age. Elevated concentrations of UA may be related to alterations in the renal handling concentration of urate and dietary changes, particularly during the third trimester [7, 9].

Some studies reported no physiological changes in renal function indicators during pregnancy in women with gestational hypertension [30]. However, in our present study, we showed that both urea and Crea were significantly lower in healthy pregnant women than the RIs in healthy adult women. If pregnant women use RIs designed for healthy adult women, then pathological states of pregnancy-related diseases may be overlooked. Therefore, the differences caused by physiological changes during pregnancy should be fully considered when establishing such RIs.

One of the strengths of our study is that it is features a longitudinal design and analyzes the same cohort of women at all time points. Compared with cross-sectional studies, longitudinal studies are more suitable for studying temporal changes of physiological parameters over different gestational ages. In addition, we used strict inclusion and exclusion criteria to ensure that the enrolled pregnant women all conceived naturally, experienced uncomplicated pregnancies, and delivered healthy singleton newborns. Furthermore, RI calculations were performed according to the IFCC and appropriate statistical analysis of reference values. Longitudinal studies only require half of the sample size required for cross-sectional studies in order to estimate gestational age-specific percentiles with the same level of accuracy. Furthermore, the number of participants included in our present research conformed with the minimum standards set by the IFCC. Clinical studies, involving a large number of participants, are now needed to verify the RIs of pregnant women.

## Conclusions

In this study, we confirmed that pregnancy results in changes in ten laboratory blood tests for serum lipid levels and renal function indices. More importantly, we conducted appropriate RIs for different gestational periods, and measured the proportion of pregnant women that would be misclassified using RIs established for healthy women. It is vital that we establish RIs for blood indicators to be used in different periods of a normal pregnancy.

## Supplementary Information

**Additional file 1: Supplemental table 1.** Correlation relationships between BMI, weight gain in pregnancy and serum lipid level.

## Data Availability

The datasets generated during and/or analyzed during the current study are available from the corresponding author on reasonable request.

## Abbreviations

Apo-A1: Apolipoprotein-A1
Apo-B: Apolipoprotein-B
BMI: Body mass index
Crea: Creatinine,
Cys-C: Cystatin C
HDL-C: High density lipoprotein cholesterol
LDL-C: Low-density lipoprotein cholesterol
GFR: Glomerular filtration rate
RIs: Reference intervals
TC: Total cholesterol
TG: Triglyceride
UA: Uric acid
VLDL: Very low-density lipoprotein

## References

[1] Teasdale S, Morton A (2018). Changes in biochemical tests in pregnancy and their clinical significance. Obstet Med.

[2] Soma-Pillay P, Nelson-Piercy C, Tolppanen H, Mebazaa A (2016). Physiological changes in pregnancy. Cardiovasc J Afr.

[3] Cheung KL, Lafayette RA (2013). Renal physiology of pregnancy. Adv Chronic Kidney Dis.

[4] Harel Z, McArthur E, Hladunewich M, Dirk JS, Wald R, Garg AX (2019). Serum creatinine levels before, during, and after pregnancy. JAMA.

[5] Friis PJ, Friis-Hansen LJ, Jensen AK, Nyboe AA, Lokkegaard E (2019). Early pregnancy reference intervals; 29 serum analytes from 4 to 12 weeks' gestation in naturally conceived and uncomplicated pregnancies resulting in live births. Clin Chem Lab Med.

[6] Larsson A, Palm M, Hansson LO, Axelsson O (2008). Reference values for clinical chemistry tests during normal pregnancy. BJOG.

[7] Dai Y, Liu J, Yuan E, Li Y, Wang Q, Jia L (2018). Gestational age-specific reference intervals for 15 biochemical measurands during normal pregnancy in China. Ann Clin Biochem.

[8] Klajnbard A, Szecsi PB, Colov NP, Andersen MR, Jorgensen M, Bjorngaard B (2010). Laboratory reference intervals during pregnancy, delivery and the early postpartum period. Clin Chem Lab Med.

[9] Jia L, Yongmei J, Leiwen P, Yi Y (2017). The reference intervals for renal function indexes in chinese pregnant women. Pak J Pharm Sci.

[10] Wayne, PA (2008). Defining, Establishing, and Verifying Reference Intervals in the Clinical Laboratory; Approved Guideline—Third Edition. CLSI document C28–A3.

[11] Robinson HP, Sweet EM, Adam AH (1979). The accuracy of radiological estimates of gestational age using early fetal crown-rump length measurements by ultrasound as a basis for comparison. Br J Obstet Gynaecol.

[12] Levey AS, Stevens LA, Schmid CH, Zhang YL, Castro AR, Feldman HI (2009). A new equation to estimate glomerular filtration rate. Ann Intern Med.

[13] Solberg HE (2004). The IFCC recommendation on estimation of reference intervals. The RefVal program. Clin Chem Lab Med.

[14] Ministry of Health PRC. Reference intervals for common clinical biochemistry tests — Part5: Serum urea and creatinine. WS/T404.5–2015.

[15] Ministry of Health PRC. Mearuements of serum high density lipoprotein cholesterol. WS/T 410–2013.

[16] Ministry of Health PRC. Measurement of serum low density lipoprotein cholesterol. WS/T 463–2015.

[17] Knopp RH, Warth MR, Charles D, Childs M, Li JR, Mabuchi H (1986). Lipoprotein metabolism in pregnancy, fat transport to the fetus, and the effects of diabetes. Biol Neonate.

[18] Aguilar CM, Baena GL, Sanchez LA, Guisado BR, Hermoso RE, Mur VN (2015). Triglyceride levels as a risk factor during pregnancy; biological modeling; systematic review. Nutr Hosp.

[19] Lippi G, Albiero A, Montagnana M, Salvagno GL, Scevarolli S, Franchi M (2007). Lipid and lipoprotein profile in physiological pregnancy. Clin Lab.

[20] Herrera E, Ortega-Senovilla H (2014). Lipid metabolism during pregnancy and its implications for fetal growth. Curr Pharm Biotechnol.

[21] Ghodke B, Pusukuru R, Mehta V (2017). Association of lipid profile in pregnancy with preeclampsia, gestational diabetes mellitus, and preterm delivery. Cureus.

[22] Wang C, Zhu W, Wei Y, Su R, Feng H, Hadar E (2017). The associations between early pregnancy lipid profiles and pregnancy outcomes. J Perinatol.

[23] Ying C, Yue C, Zhang C, Li X (2015). Analysis of serum lipids levels and the establishment of reference intervals for serum lipids in middle and late pregnancy. Zhonghua Fu Chan Ke Za Zhi.

[24] Ghio A, Bertolotto A, Resi V, Volpe L, Di Cianni G (2011). Triglyceride metabolism in pregnancy. Adv Clin Chem.

[25] Rader DJ (2006). Molecular regulation of HDL metabolism and function: implications for novel therapies. J Clin Invest.

[26] Ridker PM, Rifai N, Cook NR, Bradwin G, Buring JE (2005). Non-HDL cholesterol, apolipoproteins A-I and B100, standard lipid measures, lipid ratios, and CRP as risk factors for cardiovascular disease in women. JAMA.

[27] Mach F, Baigent C, Catapano AL, Koskinas KC, Casula M, Badimon L (2020). 2019 ESC/EAS Guidelines for the management of dyslipidaemias: lipid modification to reduce cardiovascular risk. Eur Heart J.

[28] Pottel H, Vrydags N, Mahieu B, Vandewynckele E, Croes K, Martens F (2008). Establishing age/sex related serum creatinine reference intervals from hospital laboratory data based on different statistical methods. Clin Chim Acta.

[29] Liu D, Li C, Huang P, Fu J, Dong X, Tang Y (2020). Serum levels of uric acid may have a potential role in the management of immediate delivery or prolongation of pregnancy in severe preeclampsia. Hypertens Pregnancy.

[30] Lopes VBV, van Gansewinkel T, de Haas S, Spaan JJ, Ghossein-Doha C, van Kuijk S (2019). Maternal kidney function during pregnancy: systematic review and meta-analysis. Ultrasound Obstet Gynecol.

