# Supplementary material for: Consecutive reference intervals for biochemical indices related to serum lipid levels and renal function during normal pregnancy
Source: BMC Pregnancy Childbirth. 2022 Aug 15;22:642. doi: 10.1186/s12884-022-04960-0 (PMC9377122; doi:10.1186/s12884-022-04960-0)
Supplement: Supplementary file 1 — Additional file 1: Supplemental table 1. Correlation relationships between BMI, weight gain in pregnancy and serum lipid level. [file 12884_2022_4960_MOESM1_ESM.docx]

Supplemental table 1. Correlation relationships between BMI, weight gain in pregnancy and serum lipid level.

| **Correlation** | **Gestational age** | | | | | | | | |
| --- | --- | --- | --- | --- | --- | --- | --- | --- | --- |
|  | **≤8W** | **8W^+1^-12W** | **12W^+1^-16W** | **16W^+1^-20W** | **20W^+1^-24W** | **24W^+1^-28W** | **28W^+1^-32W** | **32W^+1^-36W** | **36W^+1^-40W** |
| **BMI-TC** | 0.116 | 0.241* | 0.165 | 0.189* | -0.033 | -0.003 | -0.027 | -0.069 | 0.009 |
| **BMI-TG** | 0.352* | 0.347* | 0.321* | 0.296* | 0.252* | 0.222* | 0.195* | 0.139 | 0.158 |
| **BMI-HDL-C** | -0.350* | -0.199* | -0.192* | -0.191* | -0.261* | -0.099 | -0.053 | -0.083 | -0.111 |
| **BMI-LDL-C** | 0.247* | 0.307* | 0.203* | 0.192* | 0.005 | -0.027 | -0.072 | -0.091 | -0.070 |
| **BMI-Apo-A1** | -0.226* | -0.115 | -0.008 | -0.044 | -0.045 | 0.025 | 0.038 | 0.004 | 0.095 |
| **BMI-Apo-B** | 0.262* | 0.273* | 0.264* | 0.284* | 0.127 | 0.124 | 0.031 | 0.034 | 0.042 |
| **Weight gain-TC** | 0.058 | 0.141 | 0.100 | 0.132 | 0.113 | 0.072 | 0.079 | 0.036 | 0.053 |
| **Weight gain-TG** | 0.221* | 0.242* | 0.221* | 0.226* | 0.207* | 0.209* | 0.164 | 0.191* | 0.086 |
| **Weight gain-HDL-C** | -0.143 | -0.01 | -0.108 | 0.004 | 0.016 | -0.071 | -0.012 | -0.083 | -0.095 |
| **Weight gain-LDL-C** | 0.133 | 0.126 | 0.149 | 0.097 | 0.105 | 0.051 | 0.100 | 0.046 | 0.035 |
| **Weight gain-Apo-A1** | -0.024 | 0.085 | 0.076 | 0.070 | 0.223* | 0.117 | 0.034 | 0.008 | 0.082 |
| **Weight gain-Apo-B** | 0.125 | 0.045 | 0.153 | 0.175 | 0.200* | 0.144 | 0.152 | 0.142 | 0.118 |

**P*< 0.05 was considered statistically significant
